# Interfacing sensory input with motor output: does the control architecture converge to a serial process along a single channel?

**DOI:** 10.3389/fncom.2013.00055

**Published:** 2013-05-09

**Authors:** Cornelis van de Kamp, Peter J. Gawthrop, Henrik Gollee, Martin Lakie, Ian D. Loram

**Affiliations:** ^1^School of Healthcare Science, Institute for Biomedical Research into Human Movement and Health, Manchester Metropolitan UniversityManchester, UK; ^2^School of Engineering, University of GlasgowGlasgow, UK; ^3^Melbourne School of Engineering, The University of MelbourneMelbourne, VIC, Australia; ^4^School of Sport and Exercise Sciences, University of BirminghamBirmingham, UK

**Keywords:** modularity, motor control, intermittent control, posture, redundancy

## Abstract

Modular organization in control architecture may underlie the versatility of human motor control; but the nature of the interface relating sensory input through task-selection in the space of performance variables to control actions in the space of the elemental variables is currently unknown. Our central question is whether the control architecture converges to a serial process along a single channel? In discrete reaction time experiments, psychologists have firmly associated a serial single channel hypothesis with refractoriness and response selection [psychological refractory period (PRP)]. Recently, we developed a methodology and evidence identifying refractoriness in sustained control of an external single degree-of-freedom system. We hypothesize that multi-segmental whole-body control also shows refractoriness. Eight participants controlled their whole body to ensure a head marker tracked a target as fast and accurately as possible. Analysis showed enhanced delays in response to stimuli with close temporal proximity to the preceding stimulus. Consistent with our preceding work, this evidence is incompatible with control as a linear time invariant process. This evidence is consistent with a single-channel serial ballistic process within the intermittent control paradigm with an intermittent interval of around 0.5 s. A control architecture reproducing intentional human movement control must reproduce refractoriness. Intermittent control is designed to provide computational time for an online optimization process and is appropriate for flexible adaptive control. For human motor control we suggest that parallel sensory input converges to a serial, single channel process involving planning, selection, and temporal inhibition of alternative responses prior to low dimensional motor output. Such design could aid robots to reproduce the flexibility of human control.

## Introduction

In the everyday acts of standing and movement, humans easily generate complex multi-joint behavior. When performed in conjunction with secondary task requirements (e.g., reaching to grasp an object) the Central Nervous System (CNS) is confronted with an impressive coordination and control problem involving redundancy at many levels (e.g., sensory, biomechanical, and neuromuscular). Despite the numerous possibilities, task performance is characterized by remarkable regularity and low-dimensionality in motor output (Latash et al., 2007). Equally, performance shows “repetition without repetition” (Bernstein, 1967) which means that repetitive solutions are never the same but always vary. Open, exciting questions for researchers studying human motor behavior include: how is this abundant, robust control achieved, and how might it be replicated artificially?

In the example of human standing, multi-elemental motor outputs are defined based on an input specifying what, out of multiple task possibilities, the system has to produce as a whole (cf. Latash, 2010). This input-output coupling is faced with the “problem of selection” that is: how do accumulated task possibilities and sensory information, supplied initially through parallel channels including different modalities and sensory cells within single modalities, converge to parallel motor output? It has been suggested that at the motor level, flexibility, versatility, and adaptability in (parallel) muscle output can be achieved through modularity of the control architecture [e.g., through muscle modes, motor primitives, pattern generators, etc.; for an overview see: Latash et al. (2007), Safavynia and Ting (2012)]. What is unknown, however, is how the many-to-many convergence from parallel sensory input to parallel motor output is organized that is, the interface of sensing and action associated with task selection.

The “problem of selection” has been studied in the context of the multiple degrees of freedom (DOFs) problem (i.e., selecting a solution from the range of possible solutions). One approach that of motor synergies, has been defined as a neural organization that ensures a one-to-many mapping between important and inconsequential variables/quantities providing for both stability of important performance variables and flexibility of motor patterns to deal with possible perturbations and/or secondary tasks (Latash et al., 2007). More recently, it was suggested that “all the DOFs at all levels always participate in all the tasks.” This hypothesis called the “principle of abundance” (Latash, 2012), predicts both stability and flexibility in performance. According to this principle, the CNS facilitates “families of solutions” each of which is able to solve a multiple DOF problem. These solutions emerge from the interplay between the state of the system “body + environment” and the (task) imposed constraints (cf. Hu and Newell, 2011). This means that at any level of the sensori-motor control system, behavior is defined by the laws of physics (cf. Latash, 2012). However, a fundamental question remains unanswered: what and where is the process by which tasks are selected and by which these families of solutions are reduced to unique actualizations at temporal instances, in other words the process of selection (Stepp and Turvey, 2010; Latash, 2012)?

In tasks such as human standing, the neuro-muscular-skeletal system uses sensory information to regulate its motor output. In control engineering terms this means that the feedback loop between the control system's inputs and outputs is closed. Redundancy implies that the motor system generates parallel possible goal-solutions (which include alternative equivalent motor solutions to the overall task goal) from parallel sensory input (Cisek, 2005) and that this mapping is many to one. Thus, in a full model, the interface between (all) sensory input and modular motor output should hypothetically include the processes of parallel goal-solution generation and convergence to an instantaneously unique goal-solution prior to motor output.

Within the generalized optimal control models of biological control (Li et al., 2004; Todorov, 2004; Todorov et al., 2005; Lockhart and Ting, 2007; Karniel, 2011, 2013; Safavynia and Ting, 2012) the process for solving the redundancy problem lies outside the low level feedback control loop: it lies within a response planner which provides (continuous) settings for the continuous feedback control loop. Thus, a single optimal solution is provided from many possibilities (Rosenbaum et al., 1995; Todorov et al., 2005). Although such a system is not invertible causing a problem for control methods using an inverse model to generate appropriate motor commands from desired movement outcomes, optimal control provides a unique solution to the control of multi-input systems (Goodwin et al., 2001). However, usually this scheme does not provide a full model for the interface between sensory input and modular motor output because: (1) the task level parameters including the goal, the cost function and the mapping between high level and low level variables are usually preselected and, (2) the processes of parallel goal-solution generation and choice-selection is usually omitted. According to the generalized optimal control models (Todorov, 2004; Lockhart and Ting, 2007; Safavynia and Ting, 2012), the CNS monitors a (small) number of task parameters and, using low-level controllers, performs continuous feedback of plant output according to the task goals and optimization constraints set by high-level commands. Although this popular framework transcends the “classic robotics” approach in which trajectories are planned in joint space, and subsequently executed using servo control governing joint torque, even if correct, it begs the question of the process by which the task and optimality criteria are selected.

**Figure 1:**
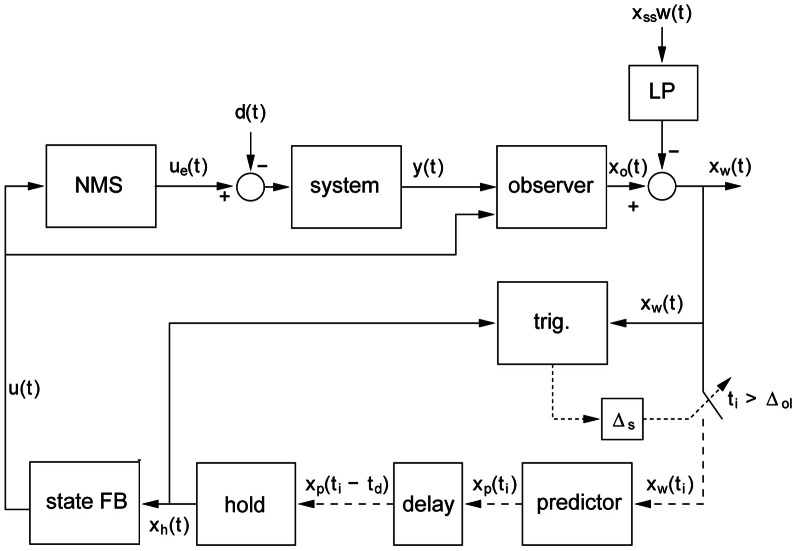
**General model of intermittent control.** The intermittent predictive controller includes continuous control as a special case, but generally the predicted system state is only used intermittently to update the time varying control signal sent from the generalized “hold” to the actuator. “Trig.” detects when the control trajectory is to be updated and this event trigger requires three conditions: (1) a single event must be detected (i.e., all events within the sampling delay (Δs) are considered as one), (2) a minimum open-loop interval (Δ_OL_) must have elapsed since the previous event and (3) an error signal must exceed a threshold. Scalar signals are represented by solid lines, vector signals are represented by dashed lines. The participant's neuro-muscular dynamics are modeled (linear) in the “NMS” block with input *u*(*t*). The linear external controlled system with output *y*(*t*) (represented by the “System” block) is driven by signals *u*_e_(*t*) and *d*(*t*) representing the externally observed control signal and the disturbance signal. The state of the composite “NMS” and “System” blocks is estimated *x*_*o*_(*t*) by the “observer” block. Sampling is preceded by an anti-aliasing low-pass filter “LP” of the subtracted set point disturbance *w*(*t*) and subject to an event delay “Δ_S_” between event and sampling. The trigger for the sampling times *t*_i_ is provided by the event detector block labeled “trig.” Sampling *x*_w_(*t*) takes place at discrete times *t*_i_. Sampled signals (represented by the dotted lines) are defined only at the sample instants *t*_i_. The future state error *x*_p_(*t*_i_) is provided by the “predictor” block. The various delays in the human controller are accounted for by a pure time delay of *t*_d_ represented by the “delay” block. The block labeled “hold” is a system-matched hold that provides the delayed version of the continuous-time signal that is multiplied by the feedback gain vector *k* (block “State FB”) to give the feedback control signal *u*(*t*). This figure and its caption are reproduced with permission from Gawthrop et al. (2011).

Recent demonstrations have shown that even for the control of external second order unstable systems^1^, continuous feedback control is not necessary, and that intermittent control has inherent advantages for control and adaptability (Loram et al., 2011). Evidence from sustained manual control of an external single degree of freedom system (Loram et al., 2012; van de Kamp et al., 2013) showed that it is unlikely that the entire sensory-motor pipeline is implemented in parallel as a continuous linear time invariant process. Rather the evidence is highly consistent with a limiting serial process along a single channel which is expressed formally in the intermittent control paradigm illustrated in Figure 1 (Gawthrop and Wang, 2011; Gawthrop et al., 2011; Gawthrop and Gollee, 2012; Loram et al., 2012). The hypothesis of a limiting serial, single channel process is supported by extensive studies in Psychology showing refractoriness in double stimulus experiments. This effect is known as the Psychological Refractory Period (PRP) (Telford, 1931; Pashler et al., 1998). Refractoriness refers to the temporal duration for which control responses cannot be, or are not modified following their initiation (Vince, 1948; Pashler et al., 1998; Gawthrop et al., 2011; Loram et al., 2012; van de Kamp et al., 2013). Extensive experimentation has firmly associated the PRP both with the Single Channel Hypothesis (Smith, 1967) and with response planning/selection in the stages of sensory analysis (SA), response planning/selection (RP/S) and response execution (RE). While SA and response execution operate through parallel channels, only the RP/S converges to a serial process along a single channel in these experimental conditions (Pashler et al., 1998).

In the current study two competing hypotheses will be tested. The multi-channel mapping hypothesis predicts continuous parallel processing which is free from refractoriness. The alternative single channel hypothesis postulates a limiting serial process along a single channel associated with the Psychological Refractory Duration (see Figure 1).

Here we aim at connecting two bodies of literature. The first concerns the PRP, the second concerns Modularity in Motor Control. We ask a novel theoretical and experimental question: namely, in the control of whole body movements, is the single channel hypothesis (characterized by a PRP) relevant for the modular organization of the motor control system? In brief, the rationale behind this question is: (1) multi-segmental control is subject to redundancy, thus the process of selection is relevant, (2) for a flexible yet integrated multi-segmental structure, organization of selection should converge along a single or coordinated number of task goals within the main feedback loop, and (3) response selection and planning is experimentally associated with refractoriness and the single channel hypothesis.

To summarize: We study control of the whole body to move the end effector (head) in accordance with a tracking target. Although the tracking task has one degree of freedom (movement of the head marker in the Anterior-Posterior plane), all the relevant joints, also when locked, have to be controlled appropriately and thus this task involves redundancy. We use our recently developed method to identify refractoriness in sustained control tasks and discriminate intermittent (serial ballistic) from continuous (parallel) control (Loram et al., 2012; van de Kamp et al., 2013). We ask:

- Is refractoriness, consistent with the single channel hypothesis, evident in this task?
- Is refractoriness and the associated serial process along a single channel relevant for modularity in motor control?
- Is there a plausible rationale for why biological control should converge to a single channel?

## Methods

### Ethical approval

The experiments reported in this study were approved by the Academic Ethics Committee of the Faculty of Science and Engineering, Manchester Metropolitan University and conform to the Declaration of Helsinki. Participants gave written, informed consent to the experiment.

### Procedure, apparatus, and measurement

**Figure 2:**
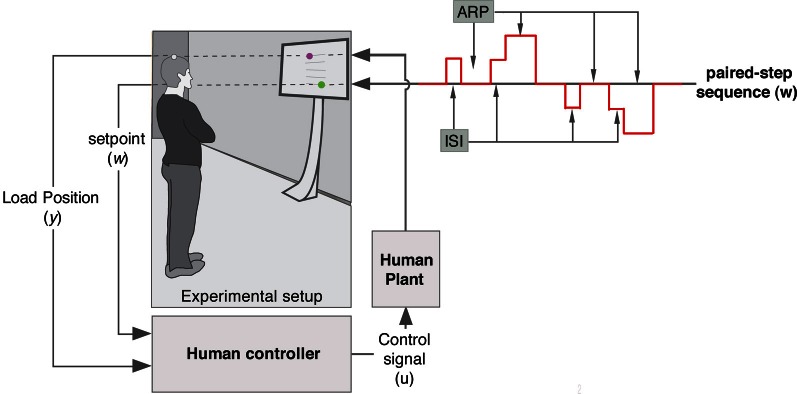
**Diagram of the experimental set up and the paired-step sequence.** The participant receives visual feed-back information about the Anterior-Posterior head position through a dot presented on a TV screen mounted on a trolley. While stabilizing posture, participants were asked to track the position of a second dot displayed on the screen. The four possible step sequences (uni- and reversed-directional step up or down) of the pursuit target are illustrated by the red line. First and second stimuli are separated by an inter-step interval (ISI).

The experimental setup is illustrated in Figure 2. Eight healthy subjects (6 male, 2 female), aged between 27 and 59 years received real-time visual feedback about the Anterior-Posterior (AP) position of a of a VICON marker, placed on the participants head whilst pursuing a double stimulus tracking sequence with varying Inter Stimuli Intervals (ISIs: 0.2, 0.3, 0.5, 0.8, 1.4, 2.4, and 4 s). Feedback was displayed on a 42″ TV screen that was mounted on a trolley positioned at a 1 m distance in front of the participant. The visual scene contained two (3 cm, green and magenta) spheres (moving up and down alongside the vertical mid line of the screen) and was constructed using the Simulink 3D Animation Toolbox (a 1 cm movement of the VICON marker in the AP direction corresponded to a 2.5 cm movement of the magenta sphere in the superior-inferior direction on the TV screen). Using Vicon's SDK we developed C++ code to stream (UDP protocol) marker data to the Simulink model that was compiled using Real-Time Workshop and executed on a laptop using Real-Time Windows Target within MATLAB v7 (MathWorks) at a sample rate of 1000 samples per second. We informed the participants that every now and then, the green target would jump up or down the screen which should be pursued by controlling the magenta sphere which represented antero-posterior head position. Participants were told to keep their feet in the initial position, to track the green target position by means of swaying their body forward and/or backwards as quickly and accurately as possible, and that, as a measure of performance, we would look at the deviation between target position and head position (i.e., the green and magenta spheres on the screen). In a randomized order, the stimuli with seven different ISIs (see above) were displayed four times. Following (van de Kamp et al., 2013), the tracking target step sequence was designed such that participants were unable to anticipate either the timing, direction, or amplitude of step change in target position (Figure 2). Unpredictability of the direction of the double step stimuli was achieved by varying the direction of the 2 cm step in target position (up-down, down-up, up-up-down to center, down-down-up to center). By varying the ISI and recovery time, also the temporal predictability was eliminated. Based on previous experiments (Loram et al., 2012; van de Kamp et al., 2013) we estimated that when stabilizing posture, a random 4–5 s period would be sufficient to recover from a step response (participants kept tracking the target which then was in the neutral/middle position). To serve as an independent base measure, the two longest ISIs were chosen in the vicinity of the recovery period. The remaining five ISI were chosen to span the range from less than to more than the expected refractory duration based on previous published work (Loram et al., 2012; van de Kamp et al., 2013). Participants verified that the delay between marker movement and its presentation on the screen (~100 ms) was not detectable. After familiarization with the intuitive control tasks and some practice all participants were ready to take part. Trial duration was approximately 4 min.

### Method of analysis

Here we applied our published three-staged method of analysis (Loram et al., 2012) to the set-point (target) and response (AP head position) signals. Details with respect to the method of analysis are stated more fully in previous work (Loram et al., 2012; van de Kamp et al., 2013) and have been restricted here to the minimum necessary.

#### Stage 1: reconstruction of the set-point

**Figure 3:**
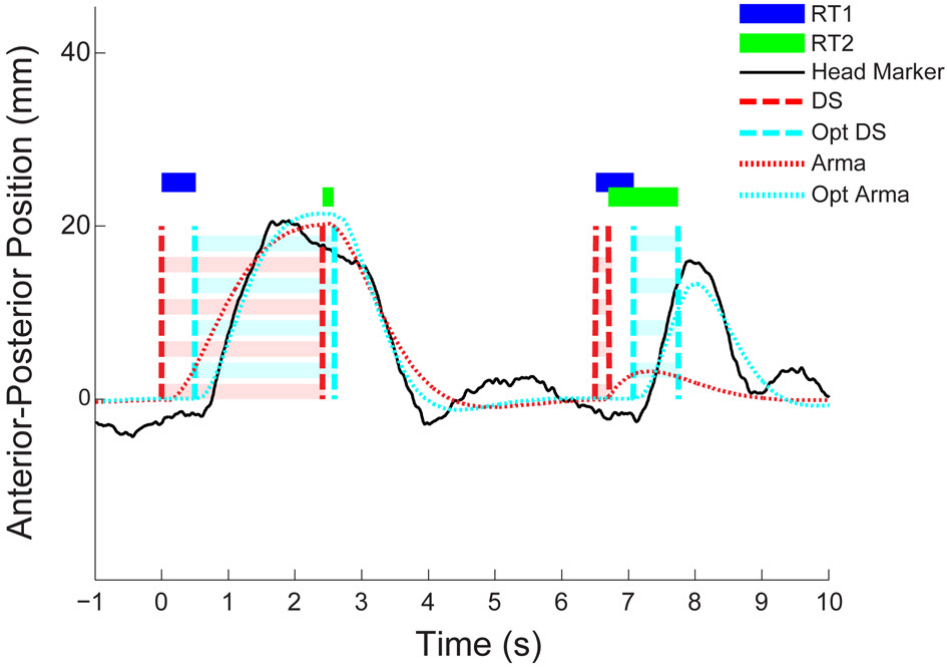
**Representative responses; reconstruction of the set-point (stage 1).** The figure shows two representative examples of a whole body response to a double-step disturbance over time (black solid lines). The first response is free of interference, the second response shows interference between responses to the second and first stimulus. The dashed line (red) shows the time-invariant optimized ARMA fit corresponding to the original/actual double step stimulus (cyan dashed line). The dotted line (cyan) shows the best fitting ARMA model corresponding to the non-time-invariant optimized step sequence. Estimates of first (RT1 in blue horizontal bar) and second (RT2 in green horizontal bar) delays hover above, and span the interval between the actual and optimized step sequence.

For each first and second step, we estimated the time delay (i.e., RT1 and RT2) between the step in target position and the subsequent whole-body-movement response (see Figure 3). This was achieved by modeling the closed loop relationship between the target (step sequence) and response (head position) as a low order, zero delay, autoregressive moving average (ARMA) process (for details see: Loram et al., 2012; van de Kamp et al., 2013). Next, the step sequence was reconstructed by sequentially and individually adjusting the instant of each step. This procedure, optimizing the fit of the ARMA model, was done in two consecutive ways; (1) reconstruction of the step sequence using a single equal adjustments of the instant of all steps -in effect determining the time delay of the ARMA model in Figure 3 and (2) reconstruction of the step sequence allowing individual adjustment of the instant of each step (i.e., the optimized ARMA in Figure 3). By optimizing the delay to each step, this “set-point reconstruction” procedure provides a distribution of response delays to first and second steps (see RT1 and RT2 in Figure 3). The statistical analysis of these delays in the second stage enables testing for refractoriness.

#### Stage 2: statistical analysis of RT1s and RT2s

To compare the distributions of RT1 and RT2 over ISIs (levels 1 through 7) a two factor (Stimulus Number and ISI) repeated measures ANOVA design is used. To evaluate significant main and interaction effects *post-hoc* ANOVAs were run. To maximize statistical power, bi-directional, and unidirectional step-pairs were analysed in one group.

The two stages outlined above allow us to test the following null hypotheses:

(i) Distributions of RT1 and RT2 are equal (i.e., a hypothesis of zero refractoriness would predict equal ranges (5–95th percentile) and means in the distributions of RT1 and RT2).
(ii) There is neither a main effect of ISI nor an interaction effect between Step Number and ISI (i.e., a hypothesis of zero refractoriness would predict that both RT1 and RT2 are independent of ISI).

If these hypotheses are rejected, the following tests provide evidence discriminating against continuous control and quantifying the extent of refractoriness in this whole body movement task.

(iii) Testing within each level of ISI for differences between RT1 and RT2 will reveal the ISI up to which there is interference between RT2 and RT1 and quantifies the duration of refractoriness.
(iv) Using linear regression to fit RT2 vs. ISI for ISIs where RT2 is significantly greater than RT1, will reveal the maximum increase in RT2 (i.e., the regression intercept (ISI = 0) minus average RT1).

#### Stage 3: model based interpretation of delays

If, in Stage 2, we find evidence of refractoriness which favors the alternative hypothesis that the single channel/IC model does apply to multi segment control of movement, the following tests would reveal its open-loop interval

(v) Repeat the regression method explained in (*iv*), assuming a least mean squares fit with slope constrained to −1 [i.e., if a response is triggered by the first step a slope of −1 is predicted in the relationship between average RT2 and ISI for ISI < open-loop interval (Pashler et al., 1998; Gawthrop et al., 2011)].

## Results

### Representative pursuit tracking responses to double step stimuli while stabilizing posture; reconstruction of the set-point (stage 1)

Figure 3 shows the comparison between a response pair without interference followed by a response pair that shows evidence of interference (i.e., the response to the second stimulus is delayed following close temporal proximity of the first stimulus). When the ISI is relatively long (see 2.4 s example Figure 3), the response delay to the second steps is not elongated relative to the first response. However, with a small ISI (see 0.2 s example Figure 3), RT2 is clearly elongated compared to RT1. This observation is quantified by means of the “set-point reconstructed” ARMA procedure. That is, if in Figure 3 we overlay the participant's whole body response (in solid black), the ARMA prediction in dotted red, and the “set-point reconstructed” ARMA prediction in dotted cyan we see that reconstructing the set-point results in a better (ARMA) description of the data. If we then compare the delays identified in this first stage of the method of analysis (i.e., the blue and green bars in Figure 3, displayed over the interval between the actual and optimized steps) we see that for the small ISI, RT2 is elongated relative to RT1.

### Statistical analysis (stage 2): group results

**Figure 4:**
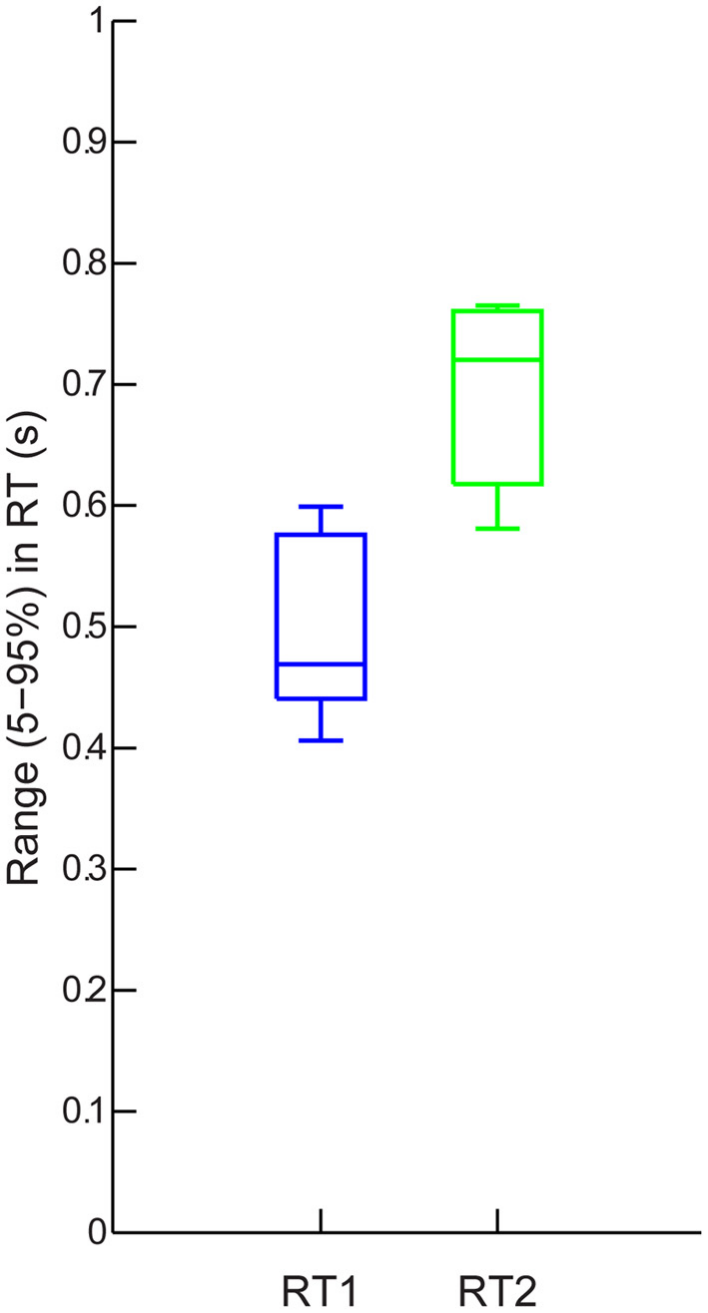
**Group results: Ranges (5–95%) of delays in RT1 (blue) and RT2 (green).** Each box shows, the median range (central mark), the 25 and 75th percentile range (the edges of the box are), and the most extreme data points not considered outliers (the whiskers) of these ranges combined across the eight participants.

Figure 4 shows that the average 5–95% range in RT was systematically affected by Step Number. The mean range in RT was significantly higher for step 2 than for step 1 [693 ± 77 ms, 497 ± 75 ms, *F*_(1, 7)_ = 48.8, *p* < 0.0005].

**Figure 5:**
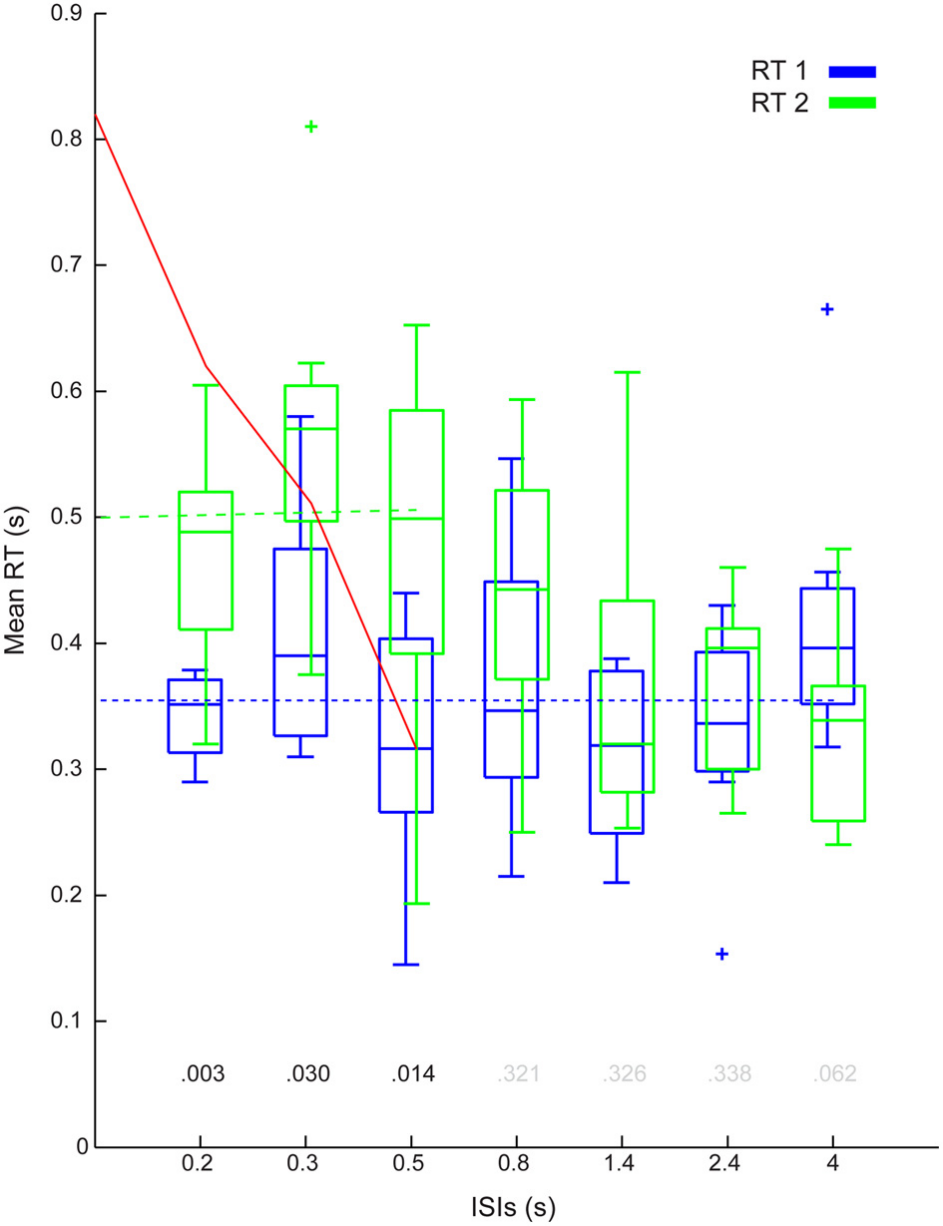
**Group results: Mean delays (stage 2).** Figure shows the inter participant mean RT1 (blue) and RT2 (green) against ISI combined across the eight participants. The *P*-values of the ANOVA's *post-hoc* test are display above each ISI level (black if <0.05, gray if not). The blue dotted line shows the mean RT1, the dashed green line shows the unconstrained regression linear fit between (interfered) RT2 and ISIs. The red line is a linear interpretation of the single-channel hypotheses (please note that the steps between ISI do not necessary increase linearly).

The mean RT (see box plots in Figure 5) was significantly higher for step 2 compared to step 1 [431 ± 130 ms, 357 ± 95 ms, *F*_(1, 7)_ = 14.2, *p* < 0.01]. Combining RT1s and RT2s showed a significant increase in RT with decreasing ISIs [406 ± 93 ms, 488 ± 135 ms, 397 ± 149 ms, 403 ± 117 ms, 339 ± 103 ms, 349 ± 79 ms, 375 ± 103 ms, *F*_(6, 42)_ = 3.83, *p* < 0.05]. The significant interaction effect between Step Number and ISI, [*F*_(6, 42)_ = 3.19, *p* < 0.05] indicates that reducing the ISI had different effects on RT1 compared to RT2. Conducting two separate *post-hoc* tests to break down the interaction, showed a significant effect of ISI on the RT2s, [*F*_(6, 42)_ = 4.53, *p* < 0.05], but not on the RT1s.

Refractoriness was quantified in three ways. The first metric, as shown in Figure 5, revealed that RT2 was increased relative to RT1 for ISIs up to 500 ms (see Figure 5 for *p*-values of the planned comparison of Step Number at each ISI). The second metric showed a mean increase in RT2 of 145 ms [subtracting the average RT1 (355 ms) from the intercept (500 ms) of the regression line of mean interfered RT2s over ISIs (cf. Figure 5)].

### Single channel interpretation of RT interference (stage 3)

**Figure 6:**
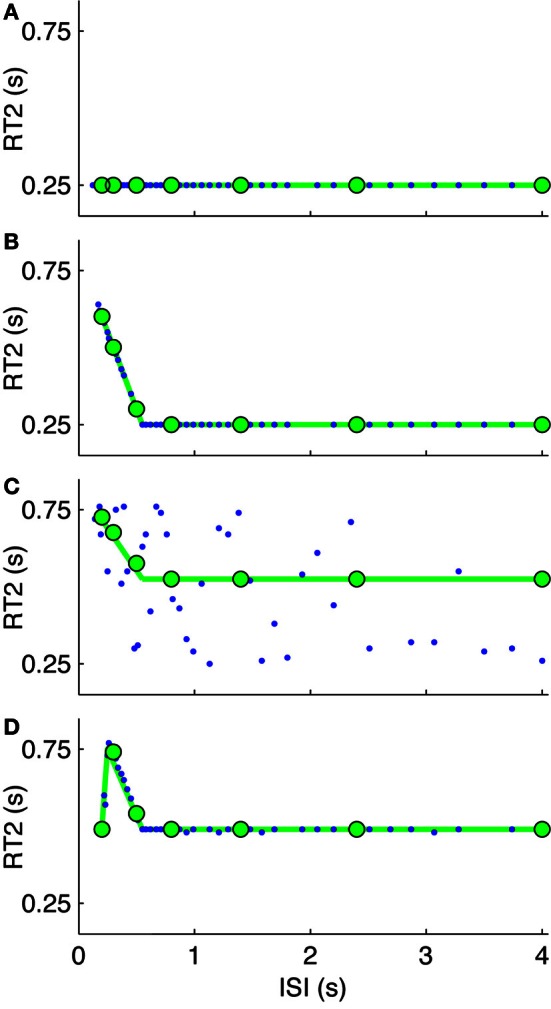
**Model based interpretation (stage 3).** Parameter variants from the generalized IC model of Figure 1 showing several possible relationships between RT2 and inter-step interval (ISI) indicative of serial ballistic (intermittent) and continuous control behavior. The simulated system is zero order. The open-loop interval (Δ_OL_) is 0.55 s and feedback time delay (*t*_d_) is 0.25 s. For four models: **(A)** continuous LTI (Δ_OL_= 0), **(B)** externally-triggered intermittent control with a prediction error threshold, **(C)** internally-triggered intermittent control (with zero prediction error threshold, triggered to saturation), and **(D)** externally-trigger intermittent control supplemented with a sampling delay of 0.25 s which us associated with the ISI at the maximum delay for RT2. The joined green circles represent the theoretical delays as a function of ISI which are confirmed by the model simulations (blue dots). This figure and its caption are based on van de Kamp et al. (2013).

The red line in Figure 5 (as discussed in Loram et al., 2012; van de Kamp et al., 2013) represents the mean RT2s in accordance with the Single Channel interpretation for the externally triggered Intermittent Control model in Figure 6B. The intercept of the red line (820 ms) in Figure 5 minus the base line of the refractory duration (i.e., the average RT1 355 ms) provided a third metric (465 ms) for the refractory duration. Like we showed in van de Kamp et al., 2013, this third metric corresponded closely to the first metric (compare 465–500 ms) both transcending the second, unconstrained linear regression, metric (i.e., 145 ms).

## Discussion

### Summary of results

In this study we hypothesized that refractoriness is relevant to the coordination of multi-segmental movement. Eight participants controlled their whole body to ensure a head marker tracked a target as fast and accurately as possible. The following results were shown unambiguously.

- Refractoriness was present in whole body movement. Delays in response to the first step (RT1) were independent of the inter-step interval (ISI). Delays to the second step (RT2) depended on ISI were greater than RT1 for ISI's less than and including 500 ms.
- The refractory duration was substantial. The ISI up until which there was distinct interference between responses, was 500 ms. The delay to RT2 compared with the mean delay for RT1 was 200 ms at an ISI of 0.3 s
- At the smallest ISI, the relationship between RT2 and ISI departed from the linear relationship between RT2 and ISI predicted by the single channel hypothesis.

This paper concerns evidence for refractoriness, the relevance of refractoriness for modularity in motor control, and the possible rationale for a serial process along a single channel. Following the facts established here and previously (van de Kamp et al., 2013) we discuss the following issues:

- Is this evidence of refractoriness consistent with a serial process along a single channel?
- What is the relationship between intermittent control and modularity of motor output?
- Is there a possible rationale for why biological control should converge to a serial process along a single channel?
- Is there any plausible neural substrate for a central, serial, single channel process?
- Could the design of autonomous robots benefit from a module including a serial, single channel process?

#### Is this evidence of refractoriness consistent with a serial process along a single channel?

The evidence for refractoriness in this whole body movement task is clear even with a relatively small sample of trials and participants. This evidence of increased delays for RT2 at low ISI is consistent with sustained manual control of an external, second order, single degree of freedom system (van de Kamp et al., 2013). The similarity includes the evidence that the increased delay for RT2 vs. RT1 is reduced at the smallest ISI. The current results demonstrate refractoriness in sustained movement control of the whole body. This result extends the relevance of refractoriness in sustained control beyond manual tracking where it might be argued that control of the hand is more refined and specialized than control of the postural muscles in the legs and trunk. This result also extends the relevance of refractoriness beyond control of a uniaxial joystick the task in our previous work (Loram et al., 2011, 2012; van de Kamp et al., 2013) because this whole body task requires coordinated control of multiple kinematic segments. Our results appear contradictory to the current prevailing hypotheses of optimal feedback control in which feedback proceeds continuously along low level feedback loops in which the goal and strategy are preset. Thus, the interpretation we have made previously (van de Kamp et al., 2013) applies also to this task of moving the whole body to control head position and the reader is referred to that discussion. The key point is that refractoriness is not compatible with a continuous, time invariant and linear process and that because the system is refractory, redundant, time varying with sensory delay and containing many DOFs, a serial process along a single channel is relevant to the control of such a system. The reader will probably not be surprised that human motor control is non-linear. However, the finding of refractoriness, a systematic time variance in which responses show increased delay when they follow a closely preceding response, points to a modular element or process in the human motor control architecture that authors usually neglect.

**Figure 7:**
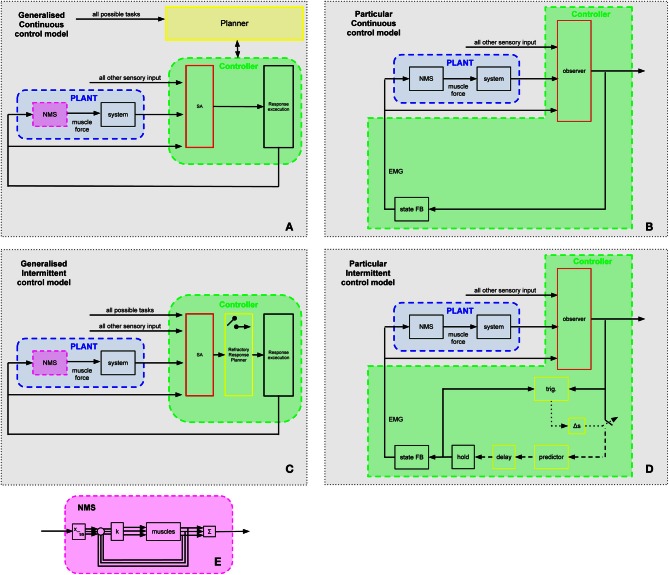
**Generalized control schemes.** In the Continuous Optimal Feedback scheme (panel **A**), task selection occurs at the “Planner” level ordering the selected strategy to be employed continuously via the low level feedback mechanism. This feedback loop consists of the “Controller” enclosing the continuous stages of sensory analysis (SA) and response execution (RE). Panel **B** shows a particular version of panel **A**: a standard engineering controller with observer and state FB blocks. The command signal serves as a single input to the “Plant” whose neuro-muscular system (NMS; panel **E**) synergistically (e.g., pattern generators, muscle modes, synergies, or optimal feedback systems) translates it to the multiple muscles according to its current parameter settings. Once these are routed, the underlying muscle forces actuate the multi-segmental system in the space of the elemental variables. In the generalized Intermittent Control scheme (panel **C**), the IC model (i.e., the “refractory Response Planner” from Figure 1) forms the intermediate stage between sensory analysis (SA) and response execution (RE); an online process of selecting one movement alternative from the many possible which occurs within the feedback loop that regulates the task. Panel **D** shows a particular version of panel **C** based on the authors' implementation of intermittent control in engineering terms (Gawthrop et al., 2011, Figure 2). Panel **E** shows a particular hierarchical representation of the NMS block of panels **A**–**D** where *k* is the feedback gain, Sigma generates a weighted sum of muscle forces and x__ss_ synergistically allocates the desired forces to each muscle.

Does the evidence of refractoriness imply a serial process along a single channel? If RT2 were linearly related to ISI with a gradient of −1, then as clearly articulated by Pashler and Johnston (1998) that would be consistent with a serial process along a single channel in which a second process cannot start until a first process has completed. Within a control system, this idea is represented within an intermittent control paradigm in which feedback cannot be applied until a minimum open loop interval has elapsed (Figure 1) (Gawthrop and Wang, 2009, 2011; Gawthrop et al., 2011; Gawthrop and Gollee, 2012; Loram et al., 2012). The minimum open loop interval, or intermittent interval as it is called, is an implementation of the single channel PRP as expressed by Craik (Vince, 1948) and Pashler (Pashler et al., 1998). Within this event driven intermittent control paradigm (Figures 1, 7), elapse of the minimum open loop interval is one of the two necessary conditions for triggering a feedback informed control trajectory. This ensures that control proceeds serially as a sequence of control actions that are constrained to be ballistic for at least the minimum open loop interval. This serial process along a single channel, and the associated refractoriness, is not represented by state related switching in which a state dependent error signal crosses a threshold. State related triggering is advocated by some authors (Asai et al., 2009; Suzuki et al., 2012) and is represented in the second triggering condition of our intermittent control paradigm (Figure 1).

The purpose of the model simulations in Figure 6 is to show that: (1) a continuous model does not show refractoriness, (2) a gradient of RT2 vs. ISI shallower than −1 is compatible with the single channel hypothesis, and (3) a reduced RT2 at the lowest ISI (i.e., departure from the red line in Figure 5) is compatible with the IC model by using a sampling delay. As illustrated in Figure 6, our model simulations show that the largest ISI at which RT2 is significantly larger than RT1 gives the open loop interval and that that the ISI at which the RT2 is largest shows the sampling delay.

Departure from the linear relationship between RT2 and ISI at small ISI or a gradient of −1 does not invalidate the single channel hypothesis (Figure 6). For this explicitly single channel model (Figures 1, 7), when one events is triggered for each target step, following the minimum open loop interval, the gradient is −1 (Figure 6B). If additional events are subsequently triggered, by an error signal crossing a threshold (e.g., due to increased noise) the slope will be less than −1. Setting the event threshold to zero so events trigger internally at the maximal possible rate will result in a gradient of −0.5 and a range of reaction times equal to the intermittent interval (Figure 6C). If applied noise is high enough, the relationship between RT2 and ISI is not defined by the IC model and the slope is zero (Loram et al., 2012). Supplementing the intermittent control model with low pass filtering of the set-point and a sampling delay (i.e., the delay between the event and the sampling instant cf. Figure 1) leads to RT2 decreasing as ISI decreases resulting in a peak in RT2 at a ISI which is equal to the sampling delay (Figure 6D). This feature reproduces the amplitude transition function (ATF) observed by Barrett and Glencross (1988a,b), in which participants combine their responses to first and second steps stimuli for small ISIs. Depending on parameter settings for noise levels, event thresholds, sampling delays, and low pass filtering; varying relationships between RT2 and ISI can be simulated consistent with the single channel hypothesis. The key evidence supporting a serial process along single channel hypothesis is the evidence of refractoriness. The decrease in RT2 with decreasing ISI at the lowest inter-stimulus-intervals may indicate incomplete convergence to a single channel for those lowest-inter-stimulus intervals (Resulaj et al., 2009).

#### What is the relationship between intermittent control and modularity of motor output?

The existence and nature of the modules in the control architecture is far from settled. For instance, regularity and low-dimensionality in the motor output are often taken as an indication of modularity but could they simply be a by-product of optimization and task constraints? Moreover, what are the relationships between modules at different levels, such as muscle synergies and basic action concepts?

Our data for this whole body tracking task and for the visuo-manual tasks that we have studied (cf. van de Kamp et al., 2013) shows refractoriness compatible with a single channel hypothesis as embodied in our intermittent control model (Figure 1). From psychology, refractoriness of this kind has been demonstrated to be associated with response selection according to a single channel hypothesis (Pashler et al., 1998). Biological systems are characterized by redundancy at multiple levels. Many joint configurations can produce the same end effector location, many actuator/muscle activation patterns produce the same joint torque, many control/neural activation patterns and pathways can produce the same actuator/muscle activation. Our data, combined with the evidence from psychology, leads us to propose that a process of selecting one movement alternative from the many possible occurs within the feedback loop that regulates this head tracking task (cf. Figure 7).

Selection of task in the space of performance variables and translation to elemental variables seems common to many schemes. To aid discussion of this question, we show in Figure 7 two main schemes. In the first scheme (see Figures 7A,B), the redundancy problem is solved outside the main feedback loop within a planner. The planner provides hierarchically and temporally prior settings to the main feedback loop, which is continuous and parallel in nature. We regard this scheme as broadly representing the prevailing idea of biological control discussed by many authors within the continuous optimal feedback paradigm (e.g., Li et al., 2004; Todorov, 2004; Todorov et al., 2005; Lockhart and Ting, 2007; Karniel, 2011, 2013; Pruszynski and Scott, 2012; Safavynia and Ting, 2012). It has been argued that this scheme is, however, un-biological (Cisek, 2005). In the second, alternative, scheme (see Figures 1, 7C,D) we propose a new hypothesis that the redundancy problem is solved within the feedback loop in a refractory response selector. The refractory response planner continuously observes multiple sensory input and multiple possible task-goal choices. The refractory response selector converges the redundant possibilities into a single output which is communicated intermittently to the response execution process. The response execution process translates the single output synergistically to the multiple muscles according to its current parameter settings. Once these are selected, the underlying control actions in the space of the elemental variables can be achieved by lower order mechanisms such as pattern generators, muscle modes, synergies, or optimal feedback systems. This second scheme generalizes the intermittent control hypothesis presented in Figure 1.

#### Is there a possible rationale for why biological control should converge to a serial process along a single channel?

Exploitation of redundancy is biologically important. Biological systems generally exploit redundancy to improve robustness and flexibility of control (Karniel, 2011). The exploitation of all available DOFs to maximize performance and flexibility is generally a sign of skill and learning (Bernstein, 1967, page 107–108), whereas the elimination of available DOFs is usually a symptom of declining ability through age (Hsu et al., 2012), disease (Oude Nijhuis et al., 2008; Pasman et al., 2011), or fear (Adkin et al., 2002).

Utilization of redundant possibilities requires selecting one possibility from many at any instant. Fundamentally, the process is one of convergence and as stated by Cisek (2005), “the processes of motor planning appear to be inextricably entwined in the processes of decision-making.” Important questions are whether convergence should narrow to a single channel and whether convergence should lie within or outside the main feedback loop. For example choice might be exercised once in selecting one strategy which is employed continuously via low level feedback mechanisms or choice might be exercised online during execution of the task in mechanisms which allow or require choice to be exercised iteratively within the feedback loop between sensory input and motor output. We see two relevant issues, namely compatibility and optimization of coordination, and rate of implementation of selection.

Compatibility and optimization of coordination: Some human tasks are incompatible. We cannot point and make a fist; we cannot flex our knees while at the same time extending them; we cannot walk and stand still simultaneously. Some tasks are partially compatible, for example walking and pointing. To avoid simultaneous engagement in mutually incompatible tasks is obviously essential. More subtly, the selection of compatible routines and the suppression of routines which are partially incompatible or merely inappropriate must underlie skilled and economical task performance. When we perform compatible tasks simultaneously such tasks need to be fully integrated to prevent mutual interference. One way to do this is to select sequentially a compatible family of lower level modules such as pattern generators, muscle modes, and synergies which translate the command to control actions. By using a single channel, only one such compatible family is selected at one time and all others are held off. For this experiment staying upright and tracking a dot might be two independent compatible tasks, two independent incompatible tasks, or one coordinated task. Our reasoning is that optimization of coordination of two tasks (dot tracking and staying upright) by eliminating mutual interference—in effect—becomes the same thing as controlling a single task in the task-space. Hence we offer the rationale that optimization of coordination leads to unification of control into a single synergy requiring a single channel for its selection.

***Rate of implementation.*** We suggest that maintaining the response selection process within the feedback loop, maximizes the rate at which response selection can be translated to motor output as an open loop process. The alternative of placing the response selection process temporally or hierarchically outside the feedback loop reduces the rate of translating response selection to motor output because it imposes the closed loop dynamics of the feedback control loop onto the translation between response selection and motor output (cf. Figure 7).

To summarize, until the point of selection, competitive commands could be prepared in parallel as envisaged in models of decision making (Cisek, 2005; Sinha et al., 2006; Carpenter et al., 2009; Noorani et al., 2011). After selection, competitors should be suppressed (Neumann, 1996). The duration of the suppression should be sufficient for the command to be executed without interference. Convergence of parallel input to a sequential, serial process along a single channel seems the ideal solution to maximize optimal, coordinated function. The serial process involves planning, selection and temporary inhibition of competing responses prior to low dimensional motor output. The serial, ballistic nature of the process removes the obligation of closed loop dynamics from response generation. The consequences are intermittent control and refractoriness. Because all input is squeezed through a single channel this is often referred to as a “bottleneck.”

#### Is there a plausible neural substrate for intermittent control and refractoriness?

Some authors support a theory of central IC, with a short planning interval (100 ms), neurologically based in a cerebello-thalamo-cortical loop, and related to a form of physiological tremor (“movement discontinuities” or “bumps”) during slow movement (Vallbo and Wessberg, 1993; Neilson and Neilson, 2005; Bye and Neilson, 2008, 2010). However, these high frequency oscillations may simply be an effect of limb resonance (Lakie et al., 2012). Our recent evidence associates IC with longer open loop intervals (250–500 ms) related to the low bandwidth of voluntary control (Craik, 1947; Vince, 1948; Navas and Stark, 1968; Hanneton et al., 1997; Slifkin et al., 2000; Loram and Lakie, 2002; Loram et al., 2005, 2011, 2012; van de Kamp et al., 2013). The “bottleneck” associated with these longer periods of refractoriness does not occur at perceptual or motor stages of information processing, but at some central stage (Pashler and Johnston, 1998; Sigman and Dehaene, 2005). Where is it located? Some brain imaging evidence using dual tasks links have suggested frontal or pre-frontal cortical structures (Jiang and Kanwisher, 2003; Dux et al., 2006). However, Szameitat et al. (2006) have suggested that the increased activity of the prefrontal structures may represent an active task scheduling mechanism (response planning—temporal ordering of the dual tasks) rather than suggesting that this is the site of the bottleneck. It may be that the bottleneck analogy—which suggests a restriction continuously throttling the flow—leads to a search for the wrong type of mechanism. A better metaphor might be that of a conductor, who intermittently engages and suppresses sections of his orchestra. The central limitation is then the rate at which the intermittent adjustments can be made. Where in the brain is an appropriate switching mechanism which involves selective inhibition and facilitation of global motor activity (the central conductor) to be sought?

The basal ganglia are a clear possibility. They are now believed (e.g., Redgrave et al., 1999) to operate as a generic action selection system, receiving input from a broad range of other brain areas, and producing output that selects particular actions to perform. The opposing roles of the two cortical re-entrant loops (direct loop—thalamic facilitation of cortical output via *ansa lenticularis*) and the indirect loop (thalamic inhibition of cortical output via *globus pallidus pars externa*) might provide a plausible mechanism to engage and inhibit cortical outputs. Gurney et al. (2001) have suggested a basal ganglia mechanism whereby *salient* actions are selected and *promiscuous* actions are suppressed, rather like center—surround antagonism in visual processing. There is evidence that the basal ganglia are important to response selection, are associated with refractoriness and are part of the IC loop (Houk et al., 2007). These findings have been used to link the basal ganglia with computational models of IC and with neurological and behavioral deficits in decision making and action selection associated with Parkinson's disease, schizophrenia and Attention Deficit Disorder (ADD) (Houk et al., 2007). This justifies the possibility that functional deficits in Parkinson's disease including the initiation and selection of new responses, freezing, and postural rigidity might be related to deficits in the IC loop. Clearly, it would be interesting to find physiological and anatomical evidence positively linking IC to the basal ganglia. Do they work as an intermittently adjusted selector switch, and what happens when this switch is damaged by disease?

#### Could the design of autonomous robots benefit from a module including a serial, single channel process?

Robots, like humans contain redundant possibilities within a multi-segmental structure. The rationale for a serial, single channel process as necessary to optimize task selection and coordination from redundant possibilities applies equally in this case as it does for humans. Intermittent control implements a serial, single channel process as the appropriate engineering solution to control problems in which there is a time consuming online computational process (Ronco et al., 1999). When the actuators, the system being controlled and the external constraints are time invariant, then the control signal can be computed rapidly from measured quantities, the reference signal, and pre-computed parameters such as the gains of a continuous optimal controller. However, when the actuators, system and constraints are time varying then online optimization and computation of the control signal is desirable. Intermittent open loop predictive control uses an intermittently moving time horizon which allows slow optimization to occur concurrently with a fast control action. This approach allows handling of time varying systems and constraints at the expense of increased online computational requirement. Thus, intermittent control provides for a time consuming online optimization process which lies at the heart of flexible predictive control. A serial, single channel process, and its implementation through intermittent control, appears to be a valuable element missing from current schemes.

## Conclusion

Eight participants controlled their multi-segmental body to ensure their head tracked a stepwise moving target as fast and accurately as possible. This is a one degree of freedom task with control redundancy. Analysis showed enhanced delays in response to target steps with close temporal proximity to the preceding step. This evidence of refractoriness is incompatible with control as a linear time invariant process. This evidence is consistent with a single-channel serial ballistic process within the intermittent control paradigm with a substantial intermittent interval related to the bandwidth of voluntary control. A control architecture reproducing intentional control of human movement must reproduce refractoriness and provide a solution to redundancy. Albeit at this stage not an experimental -deductive conclusion we suggest that best coordination of redundant possibilities provides a rationale for why the biological control architecture might converge parallel sensory input to a serial single channel process involving planning, selection and temporal inhibition of alternative responses prior to low dimensional motor output. Intermittent control, a serial, single channel process, is designed to provide computational time for an online optimization process and is appropriate for flexible adaptive control. Such design has potential to aid robots to reproduce the flexibility of human control.

### Conflict of interest statement

The authors declare that the research was conducted in the absence of any commercial or financial relationships that could be construed as a potential conflict of interest.

## Footnotes

^1^The external second order unstable system can be thought of as an average standing human behaving as an unstable second order inverted pendulum with a time constant of ~0.9 s.
